# Meningitis Cerebro-Spinalis Epidemica

**Published:** 1871-11

**Authors:** 


### MENINGITIS CEREBRO-SPINALIS EPIDEMICA.

*Extracts from a Report on this Disease, as it occurred in* 1869, *in Nauplia, Greece, by Dr. Epaminondas Kotsonopulos, from Virchow's Archiv. für Pathol. Anatomic uPhysiol. uKlin. Medicin.*

TRANSLATED BY CH. RAUSHENBERG, M.D., ATLANTA, GA.

This disease made its appearance the first time in Nauplia, and in Greece generally, in the winter of 1868 to 1869. Dr. Pretenderis, Professor at Athens, mentions an epidemic of it as having occurred in 1843, on the island of Corfu.

Nauplia has nine thousand inhabitants, and one hundred and four cases of the disease were observed, out of which number eleven cases occurred in the garrison. In the prisons, which were by no means characterized by cleanliness or otherwise hygienic conditions, not a single case was observed—a fact so much more remarkable, as Boudin, in his "Traité de Géographie et de Statistique Médicale," also states that the prisons of Hagenau, 1841, of Metz, 1849, and of Toulon, in 1851, remained free of the disease while it was raging in those cities.

The character of the disease was very malignant, as out of one hundred and four cases, sixty-seven terminated fatally.

The mortality, considered by months, is exhibited in the following statement:

| MONTHS. | NO. CASES. | DEATHS. |
|---|---|---|
| December | 2 | 2 |
| January | 26 | 14 |
| February | 45 | 23 |
| March | 15 | 12 |
| April | 3 | 1 |
| May | 1 | 1 |
| June | 1 | 1 |
| | 93 | 59 |

Eight cases out of eleven which occurred in the garrison terminated fatally.

Articular swellings, most frequently of the joints of the hand and knee, constituted, in this epidemic, the most constant initial lesion, preceding generally the meningeal symptoms, and a uniform rose-colored congestion of the conjunctiva and eyelids the next most constant and characteristic symptom of the disease. More or less severe headache was the main complaint of the patient; the delirium was often furibund; tetanic spasms of the cervical and dorsal muscles were a constant pathognomonic occurrence. Clonic spasms were observed in a few cases of small children. Tremors of the upper extremities were very prominent in a few cases, and added much to the restlessness of the patient.

Paralysis was seldom seen in this epidemic, with exception of blepharoptosis and strabismus; paraplegia, hemiplegia and facial paralyses were never observed; vesical and rectal, and also imperfect paralyses of the extremities, in a very few cases. Psychical disturbances were common. Consciousness was generally undisturbed at the commencement, but became dimmed sooner or later, and delirium, frequently very wild, occurred in all cases. Difficulty of hearing was observed in most, deafness in several cases during the course of the disease; ulcerative keratitis three times, hypopyon twice, loss of sight once. Respiratory movements generally increased in frequency—sometimes irregular, sometimes sighing as in acute hydrocephalus; a few cases terminated fatally under the symptoms of acute œdema of the lungs. Examination of the heart revealed no abnormities. Pulse at the commencement generally from ninety to one hundred; later, sometimes fifty and sixty, towards the close, frequently one hundred and thirty per minute. Abdominal symptoms not very prominent. Tongue normal, or covered with a light white coat. Nausea and vomiting of deep-green substances a common symptom. Bowels nearly always constipated, but easily moved. No enlargement of liver or spleen. Icterus in one case only. The urine was not examined. Herpes labialis was observed in a few cases, roseola once, and also petechiæ in one fatal case. Decubitus took place in all cases of long duration.

The general appearance of the disease, as it occurred there, is thus described:

After a chill—which took place nearly in every case, and was, with a severe headache, generally the first symptom—increased temperature, with repeated vomiting of bilious substances, occurred. Tongue clean, or covered with a thin white coat, bowels constipated, great restlessness, very frequently painful tumefactions of one or two articulations. After a duration of these symptoms for about twelve hours, an intermission, complete only in a few cases and preceded by a general perspiration, is soon followed by a repetition of the same symptoms, only of greater intensity. The headache is severer, the restlessness still greater; pain in the neck and back set in; more or less stiffness in the position of the patient, often assuming the character of severe opisthotonos, is observed. Delirium, first remittent, then continuous; insomnolence, gradual loss of consciousness, difficulty of hearing, enlargement and insensibility of the pupils, injected conjunctiva, sometimes strabismus and blepharoptosis are next noticed. The originally increased temperature has now become almost normal, and the pulse has, from ninety to one hundred per minute, gone down to sixty and even fifty per minute. After an indefinite duration of these symptoms, and very frequently after wild delirium, the patient sinks into a comatose condition; the formerly accelerated respiration becomes slow, more or less stertorous, and death takes place generally on the fourth or fifth day.

A majority of cases did not show an intermission at the commencement, but assumed a continuous type, with exception of a few remittent cases.

A gradual diminution of all symptoms preceded a very slow convalescence in all favorable cases. A reasonable hope of recovery was admissible in all cases which did not terminate fatally in the first few days. Some cases, principally at the commencement of the epidemic, proved fatal in from twelve to twenty-four hours—one, that of a five-year old child, in five hours. Autopsy in this case revealed: congestion of the capillaries of the cerebro-spinal membranes, a gluey exudation covering several spots of the pia mater, increased quantity and milky turpidity of the cerebro-spinal fluid.

Other cases exhibited a protracted course, and death took place, in many instances, after two or three weeks. In most cases which terminated favorably, the course of the disease was slow, and perfect recovery very protracted.

Twice the disease was observed in the form of febris comatosa (meningitis epidemica comatosa, Tourdes.) Once it commenced with the symptoms of collapse, in the case of a peasant girl at the village of Aria, who took sick after having slept one night with wet clothes on her body. I saw her a few hours after the commencement of the disease; she was icy cold, her features shrunk, eyes sunk, pulse very small and frequent, consciousness unimpaired. I would have diagnosed the case as febris perniciosa algida, if the stiffness of the neck and a swelling of the knee-joint had not been present. She recovered after forty days' sickness, completely deaf.

Nothing definite in relation to the etiology and character of the disease could be ascertained during this epidemic. The external conditions under which it appeared throw no light on the actual cause of it.

The winter in which this epidemic occurred was one of the severest over the whole of Greece. The thermometer went down to the freezing point in Nauplia several times in December and January, and snow was general all over the country. It seems, from the fact that cerebro-spinal meningitis occurs principally during the winter, that cold weather favors its development.

All provinces of Greece were visited by the disease, independent of the great variety existing in their geological and climatic conditions, proving plainly that the surface condition and soil of the country have no influence in the development of the diseases. The epidemic lasted in Nauplia from the 25th of December, 1868, to April, 1869, as exhibited in the above tabular statement, which shows that only five cases occurred in April, May and June.

The occurrence of the disease in relation to age is exhibited in the following table:

| | | |
|---|---|---|
| In the first year of life | 2 | cases. |
| From the first to the sixth | 11 | " |
| From the sixth to the tenth | 7 | " |
| From the tenth to the twentieth | 26 | " |
| From the twentieth to the thirtieth | 19 | " |
| From the thirtieth to the fortieth | 14 | " |
| From the fortieth to the fiftieth | 5 | " |
| From the fiftieth to the sixtieth | 5 | " |
| From the sixtieth to the seventieth | 3 | " |
| In the eightieth | 1 | " |
| Total | 93 | cases. |

The most cases occurred from the tenth to the fifteenth year of life. From this it becomes obvious that the disease has a great predilection for the younger classes, because, including the eleven cases which occurred in the garrison, seventy-seven cases, out of one hundred and four, were observed in patients up to thirty years of age.

As to sex, seventy-one cases, out of one hundred and four, occurred in males.

Most of the cases occurred amongst the poorer classes, who were mostly exposed to the influences of the weather. The prisons, containing over four hundred convicts, in spite of their imperfect sanitary conditions, remained free from the disease, showing that imperfect ventilation and accumulation and decomposition of animal substances have nothing to do with its development.

What is the real cause of the disease? It appears to us, from the manner in which it exhibits itself, that the usual causes of disease do not produce it, but that a peculiar poison must give origin to it. How this is produced, and of what nature it is, remains an unsolved question for the present time.

As to the nature of the disease, we consider it that of an infection sui generis, bearing no relationship to typhoid or malarious diseases.

The epidemic appearance of the disease in sections where it never had occurred before, its occasional reappearance in the following years, and its complex of symptoms, indicating something of a general character, speak against its being considered a local inflammatory affection of the cerebral and spinal membranes; and while the most of the symptoms can be reduced to permanent morbid alterations of the nerve-centers, several and very important phenomena belonging to the disease remain, which rather must result from a primary alteration of the blood. Such are, foremost, the articular affections, which were never absent, and which must be considered as symptoms of a general infection, not as pyæmic phenomena, as some have been inclined to do, as they were observed generally amongst the first, and sometimes before the characteristic symptoms of the disease. Next to these, the constant injection of the conjunctiva, the roseola and the petechiæ are symptoms which can not be developed by an inflammation of the nerve-centers alone, and which speak for a disease of a general character.

Therefore, we take epidemic cerebro-spinal meningitis to be *a general morbid process sui generis, caused by the infection of the blood by a specific poison, whose nature is still unknown*, in which process the constant inflammatory changes in the cerebral and spinal membranes must be considered as primary morbid localizations, resulting from the peculiar crasis of the blood.

In relation to the treatment of the disease, our experience is very unsatisfactory, as we lost sixty-eight out of one hundred and four cases, exhibiting a mortality of sixty-five per cent.

Quinine was used in large doses in the first nine cases, which were taken for malignant malarious fevers, but without any effect.

Mild antiphlogosis was the principal method, which was used nearly in all cases, either alone or with other therapeutical remedies, indicated by the peculiar symptoms of each case. Severe antiphlogosis, in view of the infectious nature of the disease, was generally avoided; two cases, where venesection was practiced, terminated fatally.

A few leeches behind the ears, bloody cups along the spinal column, cold applications, and calomel internally in purgative and in refracta dosi, constituted the principal treatment. With this method, the use of narcotic remedies, according to symptomatic indications, was combined, but generally with very unsatisfactory results.

In spite of these remedies and of the usual counter-irritants, as sinapisms and vesicatories, many terminated fatally. From eighty-two cases which were submitted to this mixed treatment, only twenty-five recovered, showing a mortality of nearly seventy per cent.

These unfortunate results forced us to resort to a very severe revulsive remedy; we mean the cauterization of the spinal column by the red-hot iron, as it was used the first time in this disease by Rollet. Hasse's short sentence in Virchow's Pathologie, vol. iv., under the head of "Meningitis Cerebro-Spinalis Epidemica"—that "all counter-irritants have proven without any influence on the course of the disease; only in cases where coma developed itself rapidly, energetic cauterization along the spinal column is said to have had a good effect"—induced us to try this method of treatment. We have cauterized with an olive-shaped cautery, which was pressed not too firmly to the skin, at from four to five points of the spine from the back of the neck to the loins. From the results we have obtained by this revulsive method, we are in a situation to say to all practitioners, that the efforts of a few to banish this method of practice from the treatment of this disease are certainly erroneous. We have used this method in twenty-one cases, after mild antiphlogistic treatment, and in twelve cases with success. If we deduct from the number of fatal cases four in which cauterization was used too late, and take into consideration that it was only used in very severe cases, it becomes evident that this method should never be neglected in epidemic cerebro-spinal meningitis.

This method was used not only in commencing coma, but also while the symptoms of excitement were still going on.

In the first case, the torpor of the patient was checked by the use of the hot iron; in the other, a quiet sleep generally ensued. We are inclined to ascribe to the cauterization, besides its permanent and severe derivating effects, an exciting one, which is observed during its application. Can cauterization, by exciting the vaso-motor nerves, not produce a contraction of the blood-vessels?

A comparison of the results of our treatment without cauterization shows twenty-five recoveries out of eighty-two cases; with cauterization, twelve recoveries out of only twenty-one cases.

We need not mention, that in the later stages of the disease, during a long convalescence, a permanently tonic treatment has generally been instituted.

